# Changes in pigment, spectral transmission and element content of pink chicken eggshells with different pigment intensity during incubation

**DOI:** 10.7717/peerj.1825

**Published:** 2016-03-17

**Authors:** Yue Yu, Zhanming Li, Jinming Pan

**Affiliations:** 1 Department of Biosystems Engineering, Zhejiang University, Hangzhou, China; 2 State Key Laboratory of Soil Plant Machinery System Technology, Beijing, China

**Keywords:** Pink pigment, Light, Percent transmittance, Chicken eggshell, Element content

## Abstract

**Objective.** The objective of this study was to investigate changes in pigment, spectral transmission and element content of chicken eggshells with different intensities of pink pigment during the incubation period. We also investigated the effects of the region (small pole, equator and large pole) and pink pigment intensity of the chicken eggshell on the percent transmission of light passing through the chicken eggshells.

**Method.** Eggs of comparable weight from a meat-type breeder (*Meihuang*) were used, and divided based on three levels of pink pigment (light, medium and dark) in the eggshells. During the incubation (0–21 d), the values of the eggshell pigment (Δ*E*, *L*^∗^, *a*^∗^, *b*^∗^) were measured. The percent transmission of light for different regions and intensities of eggshell pigmentation was measured by using the visible wavelength range of 380–780 nm.

**Result.** Three measured indicators of eggshell color, Δ*E*, *L*^∗^ and *a*^∗^, did not change significantly during incubation. Compared with other regions and pigment intensities, eggshell at the small pole and with light pigmentation intensity showed the highest percent transmission of light. The transmission value varied significantly (*P* < 0.001) with incubation time. The element analysis of eggshells with different levels of pink pigment showed that the potassium content of the eggshells for all pigment levels decreased significantly during incubation.

**Conclusion.** In summary, pigment intensity and the region of the eggshell influenced the percent transmission of light of eggshell. Differences in the spectral characteristics of different eggshells may influence the effects of photostimulation during the incubation of eggs. All of these results will be applicable for perfecting the design of light intensity for lighted incubation to improve productivity.

## Introduction

During the artificial incubation of chicken eggs, five factors are well known to play important roles in embryonic development and are usually carefully controlled: temperature, humidity, partial pressures of oxygen and carbon dioxide (ventilation), and the frequency of turning eggs (Nelson et al., 2004; Portugal et al., 2014). Moreover, during natural incubation in birds, eggs would certainly receive light stimulation when birds leave for feeding, whereas in commercial incubation, complete darkness is often employed. In recent years, studies have shown that embryonic development is affected by light (Adam & Dimond, 1971; Rogers, 2008; Shafey & Al-Mohsen, 2002). Photostimulation during incubation can improve growth and hatchability (Garwood, Thornton & Lowe, 1973; Shafey & Al-Mohsen, 2002; Walter & Voitle, 1972) and decrease incubation time (Fairchild & Christensen, 2000; Ghatpande, Ghatpande & Khan, 1994; Shafey et al., 2002), thereby increasing productivity. In contrast, some reports have also indicated that photostimulation during incubation reduced or did not affect hatchability (Archer & Mench, 2014; Archer, Shivaprasad & Mench, 2009; Özkan et al., 2012a). Tamimie & Fox (1967) reported a delay in hatchability and increased incidence of embryonic abnormalities in those chicks exposed to light during incubation. However, some reports found no effect on hatchability when eggs were exposed to light during incubation (Lauber & Shutze, 1964; Zakaria, 1989).

These discrepancies might be caused by the spectral characteristics of light that reaches the embryos during incubation. Eggshell pigments differ by species and include white, pink, brown and green, and even the eggs laid by the same species can have different intensities of eggshell pigmentation (Moreno & Osorno, 2003). Comparison of untreated and manually pigmented eggshells indicated that the pigmentation of eggshells influenced the spectral transmission of light into the egg (Shafey & Al-Mohsen, 2002). Thus, the effect of light on incubation depends on the characteristics of eggs, especially of eggshells. In fact, the photostimulation effect on embryonic development depends on the amount of light that reaches the embryos (Ghatpande, Ghatpande & Khan, 1994). The spectral transmission of light is influenced by the pigment and thickness of the eggshell (Bamelis et al., 2002; Maurer, Portugal & Cassey, 2011; Shafey et al., 2005). Unfortunately, this information is still neglected during egg incubation.

Thus, the objective of this research was to investigate changes in pigment intensity, spectral transmission and element content of pink chicken eggshells with different pigment intensities during incubation. The effects of the region (small pole, equator pole and large pole) of the chicken eggshell on the percent transmission of light (**PT**) that passes through the chicken eggshells were also studied. The change of pigment, element content and percent transmission (different regions of eggshell) during incubation process were evaluated as a whole.

## Materials and Methods

All experimental protocols were approved by the committee of the Care and Use of Animals of Zhejiang University. The methods were carried out in strict accordance with the guidelines of the Association for the Study of Animal Behaviour Use of Zhejiang University.

### Grouping of eggs by eggshell pigment

Three hundred freshly laid pink fertilized eggs with approximately comparable weights, 43.5 ± 0.19 g (mean ± SEM), were obtained from a commercial meat-type breeder (*Meihuang*) flock at 52 weeks of age (Zhejiang Guangda Breeding Poultry Corporation, Jiaxing, China). The eggs were distributed among three groups according to the pigment intensity of the pink eggshell, i.e., light intensity pigment (**LIP**), medium intensity pigment (**MIP**) and dark intensity pigment (**DIP**). The pigment intensity (Δ*E*, *L*^∗^, *a*^∗^ and *b*^∗^) was measured with a color tester (CR-400, Konica Minolta, Tokyo, Japan). Three regions (small pole, equator and large pole) were measured, and the average of the values of the three regions was used for whole-egg analyses. Δ*E* represents CIE Δ*E* colour distance between the standard white plate and the sample; *L*^∗^ represents luminance or brightness of sample; a* and b* represent colour values along the red-green and yellow-blue opponent axes, respectively. The value for Δ*E* = (Δ*a*^2^ + Δ*b*^2^ + Δ*L*^2^)^1∕2^ was used Δ*E* as the standard of the classification basis, and according to the value, we defined the value of Δ*E* = 11.72 ∼ 22.49 as the LIP, Δ*E* = 22.54 ∼ 28.16 as the MIP, Δ*E* = 28.17 ∼ 41.38 as the DIP. All eggs except the samples for 0 days were fumigated with formaldehyde solution and were numbered before incubation.

### Incubation

The three groups of eggs were placed into their respective tray and then in a commercial incubator (EI-hatching, Qingdao Xingyi, Qingdao, China). The internal dimensions of the incubator were 100 cm length × 110 cm width × 95 cm height. The incubator was calibrated using a standard thermometer and hygrometer before hatching the experimental eggs. The incubator was automatically maintained at 38.0 ± 0.1 °C and 60 ± 1% relative humidity (**RH**) during the entire incubation. The turning time interval during incubation was three hours.

### Sampling and measurement

**Figure 1:**
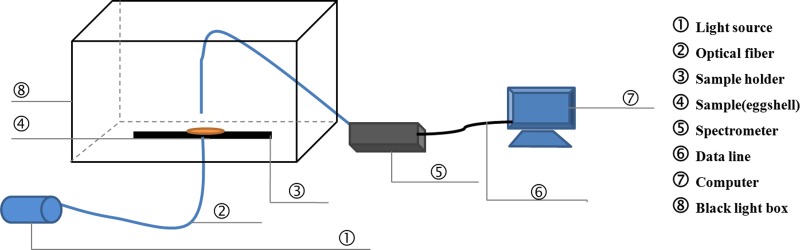
The schematic of the detection equipment for light transmission.

On days 0, 4, 8, 12, 16 and 21 during the incubation, 10 eggs were randomly sampled from each of the three trays for measurements of the chrominance difference, spectral transmission and element content of eggshells. First, the pigment intensity of three regions (small pole, equator and large pole) of each egg was measured. Then, the eggs were carefully broken, and the eggshells were washed with water and dried with paper towels. Three pieces (1–1.5 cm^2^) with the membrane intact were separated from the small pole, equator and large pole of each eggshell. The light transmission of each piece was measured using a spectrometer (QE6500, Ocean Optics, Dunedin, FL, USA). The effective detecting area was 0.25 cm^2^. The light source was a halogen lamp (HL-2000-LVP-HP 24 V, Ocean Optics, Dunedin, FL). Spectral transmission of the eggshell was recorded over the wavelength range of 380–780 nm (visible range). Figure 1 shows a schematic of the detection equipment for PT. After that, the eggshell thickness of three regions (small pole, equator and large pole) was also measured. Pieces of three regions above-mentioned were measured three times respectively to get a more accurate average value.

Finally, the eggshell samples were analyzed for potassium (**K**), sodium (**Na**), phosphorus (**P**), calcium (**Ca**) and magnesium (**Mg**) using an ICP-MS (ELAN DRC-e; PerkinElmer, Waltham, MA, USA) (Ionov, Savoyant & Dupuy, 1992). The eggshells were dried at 50 °C and then crushed with a pulverizer (Q-250B, Qijian, China) for 1 min to obtain homogeneous samples. For each group, six samples per sampling were selected for the chemical analyses; of those six samples, pairs were mixed so that there were three replicates for each sampling of each group.

### Statistical analysis

A one-way ANOVA model (SPSS 17.0) was used to test for differences among the different levels of pigment intensity and among the regions of the eggshells during incubation. Reported values represent the mean ± standard error, and the level of significance was taken as *P* < 0.05. When significance was detected, the differences between the treatment means were tested using the least significant difference (**LSD**) procedure.

## Results

### Changes in eggshell pigment during incubation

The change in the Δ*E*, *L*^∗^, *a*^∗^ and *b*^∗^ values of pink pigmented eggshells during the incubation was evaluated. Significant changes were observed in the b* value of pink pigment eggshells during incubation (Table 1). The value of b* was significantly reduced at day 4 compared with those of day 0. The value f b* represents the yellow and blue balance of the sample, with increases and decreases in the value representing more yellow and blue, respectively. However, the results of Table 1 showed no significant changes in the Δ*E*, *L*^∗^ or *a*^∗^ values of pink pigmented eggshells.

**Table 1:** The *P*-values for the effect of time on the relative change of each color indicator (Δ*E*, *L*^∗^, *a*^∗^, *b*^∗^). The Δ*E*, *L*^∗^, *a*^∗^, *b*^∗^ value of eggshell during the incubation period (0–21d).

	Incubation period (day)						
	0	4	8	12	16	21	*P*
Δ*E*	24.9 ± 1.0	26.7 ± 1.2	25.2 ± 1.0	24.8 ± 1.1	25.5 ± 1.2	27.9 ± 1.1	>0.05
*L*^∗^	76.3 ± 0.8	76.3 ± 1.1	77.4 ± 0.8	77.6 ± 0.9	77.5 ± 0.9	75.0 ± 0.9	>0.05
*a*^∗^	6.8 ± 0.3	6.8 ± 0.6	6.4 ± 0.4	6.3 ± 0.5	6.1 ± 0.5	6.2 ± 0.4	>0.05
*b*^∗^	18.9 ± 0.6^a^	21.7 ± 0.6^b^	20.6 ± 0.6^a^^b^	21.1 ± 0.7 ^b^	21.1 ± 0.7^b^	22.9 ± 1.0^b^	<0.01

**Notes.**

Values are the mean ± SEM, *n* = 30.

a, b, c, d means within a row followed by different superscripts are significantly different (*P* < 0.05).

**Table 2:** The element (K, Na, P, Ca and Mg) content (g/100 g of dry mass) of eggshells with different intensities of the pink pigment of the eggshell during incubation.

Intensity pigment	Element	Incubation period (d)						*P* value
		0	4	8	12	16	21	
LIP^a^	Na	0.099 ± 0.006	0.101 ± 0.006	0.098 ± 0.015	0.106 ± 0.008	0.098 ± 0.002	0.086 ± 0.01	>0.05
	P	0.137 ± 0.005	0.143 ± 0.019	0.144 ± 0.011	0.142 ± 0.011	0.157 ± 0.032	0.156 ± 0.026	>0.05
	Ca	39.004 ± 0.805	37.954 ± 0.734	37.759 ± 0.635	38.219 ± 0.524	38.209 ± 0.19	37.863 ± 0.608	>0.05
	K	0.047 ± 0.001	0.051 ± 0.006	0.041 ± 0.005	0.045 ± 0.003	0.046 ± 0.003	0.038 ± 0.004	<0.05
	Mg	0.454 ± 0.043	0.465 ± 0.04	0.454 ± 0.052	0.507 ± 0.031	0.492 ± 0.037	0.432 ± 0.039	>0.05
MIP^a^	Na	0.11 ± 0.008	0.104 ± 0.004	0.107 ± 0.008	0.102 ± 0.012	0.09 ± 0.004	0.091 ± 0.004	<0.05
	P	0.122 ± 0.022	0.143 ± 0.004	0.157 ± 0.006	0.153 ± 0.011	0.154 ± 0.021	0.159 ± 0.038	>0.05
	Ca	40.929 ± 1.963	38.262 ± 0.359	39.438 ± 3.033	37.301 ± 0.559	37.856 ± 0.292	38.342 ± 0.564	>0.05
	K	0.048 ± 0.002	0.051 ± 0.003	0.048 ± 0.007	0.043 ± 0.003	0.041 ± 0.002	0.04 ± 0.002	<0.01
	Mg	0.424 ± 0.042	0.451 ± 0.006	0.499 ± 0.05	0.456 ± 0.023	0.442 ± 0.041	0.447 ± 0.026	>0.05
DIP^a^	Na	0.101 ± 0.014	0.094 ± 0.003	0.096 ± 0.002	0.09 ± 0.001	0.093 ± 0.005	0.091 ± 0.008	>0.05
	P	0.136 ± 0.007	0.15 ± 0.008	0.14 ± 0.006	0.144 ± 0.019	0.145 ± 0.024	0.14 ± 0.03	>0.05
	Ca	39.186 ± 0.876	38.487 ± 0.128	37.994 ± 0.404	37.822 ± 1.215	37.814 ± 0.258	38.322 ± 0.215	>0.05
	K	0.047 ± 0.003	0.048 ± 0.004	0.043 ± 0.004	0.037 ± 0.004	0.043 ± 0.005	0.038 ± 0.005	<0.05
	Mg	0.455 ± 0.049	0.44 ± 0.021	0.446 ± 0.05	0.37 ± 0.01	0.465 ± 0.044	0.429 ± 0.045	>0.05

**Notes.**

a LIP = light intensity pigment, MIP = medium intensity pigment, and DIP= dark intensity pigment.

b Values are the mean ± SEM.

### Changes in the PT according to different regions and different eggshell pigment intensities during incubation

**Figure 2:**
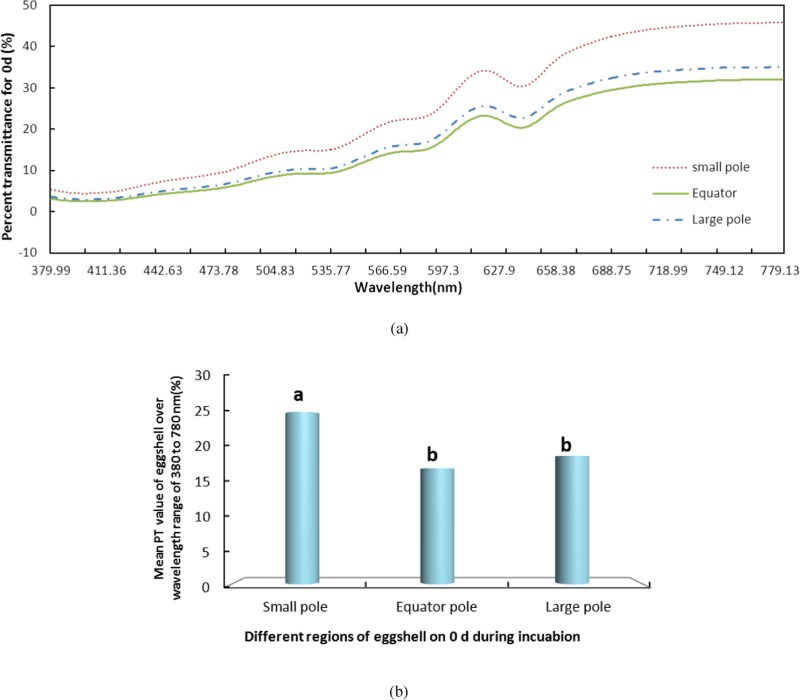
The PT of light over the wavelength range of 380–780 nm at the small pole, the equator and the large pole of pink pigment eggshells on days 0 during incubation. S, small pole; E, equator; and L, large pole. (A) shows the PT of light over the wavelength range of 380–780 nm at the small pole, the equator and the large pole of pink pigment eggshells on days 0 during incubation. (B) shows the mean PT value of eggshell over the wavelength range of 380–780 nm.

We measured the change in PT of light over the visible range at the small pole, the equator and the large pole of the eggshells on days 0, 4, 8, 12, 16 and 21 during incubation. The results of the whole period were consistent. Using day 0 (Fig. 2) as an example, the PT value of the small pole was the highest and was significantly different from those of the other regions (*P* < 0.05). The values at the equator and the large pole were 8.18% and 6.39% lower, respectively, than that of the small pole. Although the PT of the large pole was slightly higher than the equator, there was no significant difference between the large pole and the equator. The pattern of PT values at the three locations remained consistent during the entire incubation. Throughout incubation, the highest PT was observed in the small pole, followed by the equator and the large pole.

**Figure 3:**
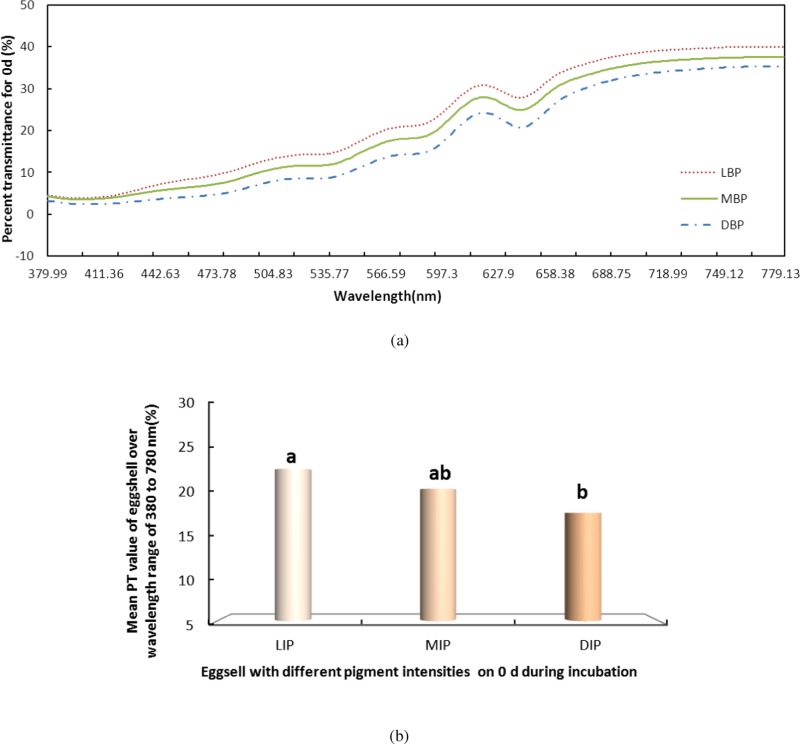
The PT of light over the wavelength range of 380 to 780 nm for the light, middle and dark levels of pink pigment in eggshells on day 0 during the incubation. LIP (L), light intensity pigment; MIP(M), medium intensity pigment and DIP(D), dark. (A) shows the PT of light over the wavelength range of 380 to 780 nm for the light, middle and dark levels of pink pigment in eggshells on day 0 during the incubation. (B) shows the mean PT value of eggshell over the wavelength range of 380 to 780 nm.

We also measured the change in PT of light over the visible range for the LIP, MIP and DIP eggshells during the entire incubation. Again using day 0 (Fig. 3) as an example, the LIP showed the highest PT of the three colour groups, and there was a significant difference among the colors (*P* < 0.05). The PT of LIP was 5.11% and 2.32% higher than DIP and MIP, respectively. The PT value for MIP was not significantly different from the other conditions. Further, the difference in PT values for the three colors of eggshells during the incubation period was the same as on day 0. The highest PT was observed in LIP eggshells, followed by MIP and then DIP eggshells. However, although the PT of MIP eggshells was higher than that of DIP eggshells, the only significant difference (*P* < 0.05) observed was between the PT values of LIP and DIP.

**Figure 4:**
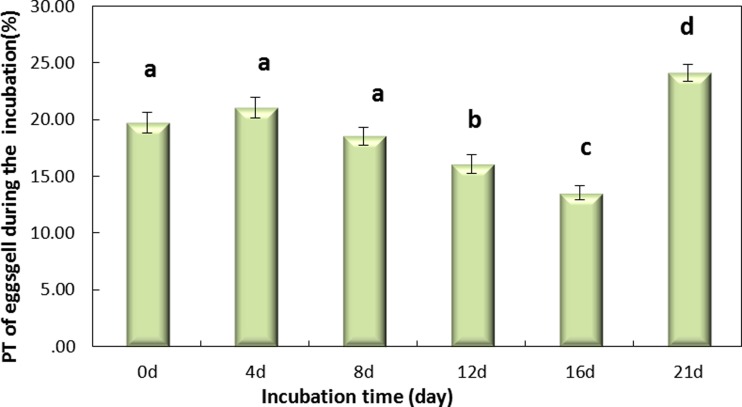
The PT(%) of light over the wavelengths of 380–780 nm at different regions of the eggshell and at different levels of intensity of the pink pigment of the eggshells at different periods during incubation. Values are the mean ± SEM, *n* = 30.

Figure 4 shows that the incubation could be separated into two periods, days 0–16 and days 16–21, according to the PT of the eggshell. The PT of eggshells increased gradually, to 13.53% on day 16 and rapidly increased to 24.12% on day 21. The trend over the whole period (0–21 day) was significant (Fig. 4, *P* < 0.001). And Fig. 4 shows that the PT of the eggshell was also significant decreased from day 0 to day 16 (Fig. 4, *P* < 0.001).

**Figure 5:**
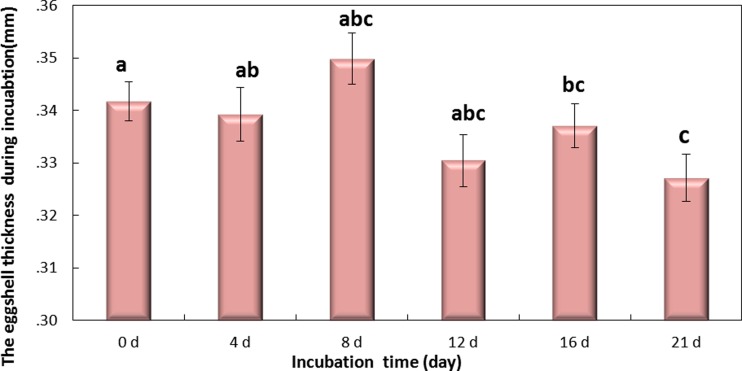
The thickness of eggshell on days 0, 4, 8, 12,16 and 21 during the incubation. If followed by different superscripts, values are significantly different (*P* < 0.05). Data were presented as mean ± SEM, *n* = 30.

### Changes in thickness of eggshell during incubation

Eggshell thickness was significantly decreased during the incubation (*P* < 0.01) (Fig. 5). The value of thickness on day 0 (0.35 mm) was significantly different from day 16 (0.331 mm, *P* < 0.01) and day 21 (0.327 mm, *P* < 0.001), and slight change was observed on day 4 (0.342 mm), day 8 (0.339 mm) and day 12 (0.337 mm) (*P* > 0.05). These data indicated that the significant decrease of eggshell thickness appeared on day 16. Theoretically speaking, the decrease of eggshell thickness may improve the PT.

### Changes in the element content of the eggshell during incubation

The concentrations of various elements, during the incubation, in eggshells with different intensities of pigment are shown in Table 2. The Ca and P contents of eggshells with different intensities of pigment did not change significantly during the incubation. However, the Na content of the MIP decreased significantly (*P* < 0.05) during the incubation, and the K content of the eggshells with all levels of pigment intensity decreased significantly during the incubation (LIP: *P* < 0.05, MIP: *P* < 0.01, DIP: *P* < 0.05).

## Discussion

Our results clearly demonstrate that the intensity of the pigment and the region of the eggshell influenced the spectral characteristics of eggshells, and these results were consistent throughout the incubation. In addition, the PT and the potassium content of the eggshells decreased as the intensity of the pink pigment increased.

Light has a profound effect on embryonic growth (Özkan et al., 2012a; Özkan et al., 2012b; Shafey, 2004; Shafey & Al-Mohsen, 2002; Shafey et al., 2005; Zhang et al., 2012). However, little information is available on the factors that cause the various changes in response to photostimulation during incubation (Archer & Mench, 2014; Rozenboim et al., 2003). When different intensities of fluorescent light were used during the incubation of eggs, the acceleration of embryonic development was dependent on the quantity of light that reached the embryos (Ghatpande, Ghatpande & Khan, 1994; Gold & Kalb, 1976; Tamimie & Fox, 1967). When pigmented and unpigmented Japanese quail eggs were placed in the same light environment during incubation, the embryonic development of the pigmented eggs was slower than that of the unpigmented eggs, which hatched earlier (Coleman & Mcnabb, 1975).

*Meihuang* birds lay pink eggs with a wide variety in the intensity of the color of the eggshell. The main pigment of pink eggshells is protoporphyrin, and the color of the pigment depends on the selective absorption of certain wavelengths of light and the reflection of others. In this study, three measured indicators of eggshell color, Δ*E*, *L*^∗^, and *a*^∗^ did not change significantly during the incubation. However, there were significant changes in the b* value of pink pigmented eggshells during incubation (Table 1). The value of *b*^∗^ was significantly different between day 0 and day 4, with the analysis on day 4 showing more yellow and therefore an increase in *b*^∗^. This change may be due to the fumigation process (all samples except those for day 0 were treated) before incubation. We added the experiment of the pigment intensity test before and after formaldehyde fumigation. As a result, only *b*^∗^ was significantly different before and after formaldehyde fumigation (Table S1). As above, there was no significant difference in eggshell color during the incubation.

However, according to part 2 of the present study, the intensity of the pink pigment of the eggshell also influenced the spectral characteristics. Light pink pigment allowed more light to pass through the eggshell than did darker pigments. Among the eggshells with different pigment intensities, the LIP eggshells had the highest PT values. Therefore, according to the results of the first two parts of the study, the incubation could cause the PT of the eggshells to decrease. Under the influence of light, the development of embryos with pigmented shells is slower than for those with unpigmented shells, and depigmentation of eggshells results in early hatching. Considering that the lower the intensity of eggshell pigment, the higher the PT, the influence of the same light intensity is different for different colors of egg, and our future research will focus on verifying this conclusion.

The PT varied among the different regions of the eggshell during the entire period of incubation, being highest at the small pole, intermediate at the equator, and lowest at the large pole. Pores are not uniformly distributed over the surface of the egg, and pore concentration varies among the regions of the eggshell (Romanoff & Romanoff, 1949). Generally, the equator and large pole have more pores than the small pole. However, the finding that the small pole of eggshell had higher PT than that of the large pole may be due to difference in the active pore area of the measured samples. A significant decrease in eggshell thickness was recorded on day 16. Theoretically speaking, the decrease of eggshell thickness may improve the PT. The slightly change before day 16 may cause no significantly PT change. The ranking order of shell components from outside to inside is shell, outer shell membrane and the inner shell membrane. At the prophase (0–7 day during incubation), shell and membrane were combined closely. The combination was decreased during the incubation process. The membrane was almost separated from the shell on day 21. A micro-gap between the shell and membrane developed step-by-step during the incubation process. Reflection and scattering caused by the micro-gap may decrease the light energy reaching the embryo, which was the reason of the decrease of PT on day 0–16. The chicks were hatched on day 21 of incubation, and the eggshells were broken and slightly destroyed, with the membrane and the shell completely separated from each other. These phenomena resulted in significantly higher eggshell PT values on day 21 than on the other days.

The element analysis of the eggshells with the different pink pigment intensities showed that the potassium content decreased significantly during incubation for eggshells with all intensities of the pigment. Na^+^ and K^+^ are important ions to retain and regulate the cell potential and osmotic pressure which are essential for living organisms. The embryo can only obtain Na^+^ and K^+^ ions from the hatching egg itself. As parts of the egg, Shell and membrane may provide Na^+^ and K^+^ ions to the embryo during incubation. This is the hypothesis for the mechanism of changes in Na and K content during incubation.

In our study, the intensity of the pigment and the region of the eggshell influenced the PT of the eggshell. Differences in the spectral characteristics of different eggshells may influence the effects of photostimulation during the incubation of eggs.

## Conclusion

In summary, the pigment intensity and the region of the eggshell influenced the PT of the eggshell. Differences in the spectral characteristics of different eggshells may influence the effects of photostimulation during the incubation of eggs. All of these results will be applicable for perfecting the design of light intensity for lighted incubation to improve productivity.

## Supplemental Information

10.7717/peerj.1825/supp-1 Supplemental Information 1 Raw data

10.7717/peerj.1825/supp-2 Table S1 The Δ*E*, *L*∗, *a*∗, *b*∗ value of eggshell before and after fumigation and values are the mean ±SEM, *n* = 30

## Additional Information and Declarations

### Data Availability

The following information was supplied regarding data availability: Raw data is available in the Supplemental Information.
