# Supplementary material for: Changes in pigment, spectral transmission and element content of pink chicken eggshells with different pigment intensity during incubation
Source: PeerJ. 2016 Mar 17;4:e1825. doi: 10.7717/peerj.1825 (PMC4806607; doi:10.7717/peerj.1825)
Supplement: Table S1 [file peerj-04-1825-s002.docx]

**Table S1.** The △E, L*, a*, b* value of eggshell before and after fumigation and values are the mean ± SEM, n=30.

| Groups | △E | L* | a* | b* |
| --- | --- | --- | --- | --- |
| Before fumigation | 26.35±1.10 | 75.19±0.89 | 7.15±0.53 | 19.59±0.57 |
| After fumigation | 26.45±1.11 | 76.39±0.99 | 6.81±0.52 | 21.37±0.58 |
| *P* | ＞0.05 | ＞0.05 | ＞0.05 | ＜0.05 |
